# Psychosocial dimensions of access and their association with contraceptive use and intention to use

**DOI:** 10.1186/s12905-023-02841-y

**Published:** 2024-01-02

**Authors:** Lonkila Moussa Zan, Clémentine Rossier

**Affiliations:** 1 ISSP, Univsersité Joseph KI-ZERBO, Ouagadougou, Burkina Faso; 2 University of Geneva, Geneva, Switzerland

**Keywords:** Cognitive dimensions, Psychosocial dimensions, Contraception, Intention to use, Side effects

## Abstract

**Background:**

Several studies suggest that psychosocial accessibility appears to be the key remaining hurdle to contraceptive use when issues of geographic and financial accessibility have been resolved. To date, the literature has considered various dimensions of psychosocial accessibility, which are not well measured by the two main sources of contraceptive data (DHS and PMA2020). In a previous paper, we have designed a framework that outlines four subdimensions of cognitive and psychosocial access and their theoretical relationship to contraceptive use and intention to use. This paper aims to study the associations between the four dimensions of access to contraception with the contraceptive use and intention to use. It also aims to explore the mediation effect of these four dimensions of access in the relationships between classical individual characteristics and contraceptive use and intention to use.

**Methods:**

The data we used came from the 6th round of the PMA2020 survey in Burkina Faso in 2018–19. This survey included 2,763 households (98.4% response rate) and 3329 women (97.7% response rate). In addition to PMA’s core questions, this survey collected data on psychosocial accessibility. Each group of questions was added to address one dimension. We use a multilevel generalized structural equation and mediation modeling to test the associations between psychosocial accessibility and contraceptive use while controlling for some individual and contextual characteristics.

**Results:**

Approval, contraceptive knowledge, and agency were associated with contraceptive use, while fears of side effects were not. Approval and agency explain part of the effects of education and parity on contraceptive use. Exposure to family planning messages had a positive impact on women’s contraceptive agency.

**Conclusion:**

FP messages can help enhance women’s contraceptive agency, and then, contraceptive use, regardless of age and parity. The analysis highlights the mediator effects of contraceptive approval and agency on the association between parity and education with contraceptive use.

## Background

Recent assessments have highlighted the importance of psychosocial obstacles to contraceptive use, particularly in settings where the demand for fertility remains high. In Burkina Faso, although women and men express the desire to space out pregnancies, they still desire large families. This is especially true of men, who are thus less committed to family planning (FP) [1] (Ministère de la Santé 2017). Most women lack autonomy of choice about health issues, and indeed, only 8% of them make independent decisions about their health care [2] (INSD and MACRO 2012). Other obstacles, such as fear of side effects (SE), appear to be widespread in the population [1] (Ministère de la Santé 2017), as they constitute 25% of the stated reasons for contraceptive discontinuation [2] (INSD and MACRO 2012).

These results contrast with earlier research findings, which established that the availability of quality services is an essential factor in contraceptive use [3] (Skiles et al. 2015). However, access to contraception, like access to health, is a multidimensional concept. A recent summary of this literature proposes to approach access to contraception through six dimensions [4] (Choi, Fabic and Adetunji, 2016). Four of these pertain to supply: geographic accessibility, service availability, administrative accessibility and affordability. These authors also note that two of those dimensions (cognitive and psychosocial access) are more relevant and challenging today than the others because, in addition to being related to contraceptive demand, they are still prevalent in low-income countries, despite substantial progress made concerning obstacles on the supply side [4] (Choi, Fabic and Adetunji, 2016). Those two dimensions can be encapsulated within the same topic of psychosocial accessibility, which we have divided into four dimensions [5] (Zan,2021).

### Dimension 1: contraceptive knowledge

This dimension encompasses knowledge of contraceptives and includes knowledge of where these contraceptives may be sourced, the mechanisms of use, associated side effects of contraceptive methods, and their management. The lack of accurate, objective, and reliable information (from formal sources) is considered the root of rumors about contraception [6–8] (Dehlendorf et al. 2020; Gilliam et al. 2004; Pazol et al. 2018). The “diffusion of innovations” theory [9] (Rogers 2010) holds that an idea will spread through populations if it is consistent with the community’s values, beliefs, and needs. This theory links the spread of formal knowledge about contraception to local sociocultural perceptions of fertility and sexuality. Indeed, in a setting that values large families and the control of sexuality within marriage, any idea that tends to discourage contraceptive use will spread easily and will be valued over information from health professionals [7] (Gilliam et al. 2004). Earlier research has already observed that the discrediting of formal knowledge about contraception and the dissemination of rumors regarding side effects must reflect social disapproval of contraception [10] (Casterline et al. 1997).

### Dimension 2: fear of side effects

The spread of rumors (informal local knowledge) depicting contraception negatively in turn increases fears of side effects, which reduces adoption among potential users or continuation among current users. Indeed, the decision to commence contraception and vigilance in contraceptive use are based on a balance between what a woman feels about contraceptive methods and what she feels about pregnancy avoidance [11] (Miller 1986). For example, people often voice their fears about the risks of primary or secondary infertility relating to the side effects of contraception when explaining their choice [12] (Sedlander et al. 2018). Ultimately, a woman in need of contraception may end up not using it if the fears of side effects supersede her motivation to avoid pregnancy. Many studies have demonstrated that fears of side effects constitute populations’ central reservations about and major barriers to the current and future use of contraceptive methods [13, 14] (Bongaarts and Bruce 1995; Sondo et al. 2001).

### Dimension 3: approval of contraception

Approval, regarded as a positive or negative assessment or valuation of a behavior [15] (Rossier and Bernardi 2009), is also important when it comes to contraceptive adoption. Many studies have found that approval is a major determinant of contraceptive use [16] (Islam and Hasan 2016). In a landmark study on several countries in sub-Saharan Africa, some scholars used DHS questions on a woman’s approval (which have since been removed) and her perception of her husband’s consent as indicators of attitudes toward FP. They concluded that “attitudinal resistance” remains a major barrier to contraceptive use [17] (Cleland, Ndugwa and Zulu, 2011). However, a study in rural and urban Burkina Faso [18] (Rossier 2007) refined the notion of approval by distinguishing between the approval of contraception in different life situations: before marriage, or birth spacing, and for birth limiting. This study found that approval was widespread for spacing in that context but uncommon before marriage and weak for limiting. This connects to the still-unfavorable social representations of premarital sexuality and the limitation of family size in this environment.

### Dimension 5: contraceptive agency

Many studies have shown that women’s autonomy, decision-making, and empowerment are associated with contraceptive use [12, 19, 20] (Bamiwuye et al. 2013; Ghose et al. 2017; Sedlander et al. 2018). Agency is defined as the ability to choose and act upon one’s choice [21, 22] (Kabeer 1999; Sapin, Spini and Widmer 2007). Women’s agency, measured through decision-making and freedom of movement, is found to be associated with women’s contraceptive use in lower- and middle-income countries [23] (James-Hawkins et al. 2018). However, agency may differ across practices: a woman may be freer to engage in socially approved practices, while stronger social control may be expected for less approved practices, such as contraceptive use. Therefore, it is necessary to measure agency not in general but specifically for contraception.

Based on the literature, there is a justification for the need to collect and analyze data on the four dimensions of psychosocial access to contraception. In this paper, we use that framework and the data collected on the four dimensions in PMA2020 Round 6 in 2019 in Burkina Faso to achieve the following three objectives. First, we will study the relationship between these varied psychosocial dimensions. Second, we describe the relationship between them and contraceptive use. Third, we will analyze the associations between those dimensions, other individual characteristics, contextual characteristics, and contraceptive use.

## Data and methods

### Data

The data are derived from Round 6 of the PMA2020 survey in Burkina Faso conducted from December 2018 and January 2019 in a nationally representative sample spread over 83 enumeration areas. We interviewed 2,763 households (98.4% response rate) and 3329 women (97.7% response rate). In addition to PMA’s core questions, this survey collected data on psychosocial accessibility. Each group of questions was added to address one dimension. Only the first question about knowledge of contraceptives is also present in the classic PMA2020 or DHS questionnaire. We included two questions to collect and adjust data on fear of side effects. We also included questions designed specifically to appraise the level of approval of contraceptive use by non-married individuals for spacing and for limiting pregnancies as well as seven questions relating to women’s ability to discuss, negotiate, and make decisions on childbearing and contraceptive use.

### Methods and variables

To summarize the data on each dimension, we computed latent dimensions [5] (Zan 2021), which were treated as continuous variables in this study. Therefore, we can use path analysis to model their relationships with contraceptive use while considering other socioeconomic and demographic variables. Structural equation modelling (SEM) [24] (Acock 2013) is recommended when more than one regression equation is required for modeling. SEM uses a set of statistical techniques that comprise a general linear model, a path model, and a Confirmatory Factor Analysis (CFA) model. These techniques allow us to test our hypothetical relations (between the four dimensions and contraceptive use) using our empirical data, as shown in the framework. In our case, we used a generalized multilevel SEM (GSEM) to fit the relationship between women’s characteristics, contextual characteristics, psychosocial dimensions, and contraceptive use. If GSEM bolsters some features that SEM does not, especially the possibility of including contextual-level variables, it has other limitations compared to SEM. The main limitation is the lack of some estimators of goodness of fit. We also used the “clustered robust” option to obtain adjusted standard errors in enumeration areas.

The women’s characteristics, such as wealth score and parity, are continuous in the models. Although educational level is an ordinal categorical variable, we considered it to be continuous in the model. The relationship between age and contraceptive behavior is not linear; we also recoded age into three categories: AGE_1: 15–24 years; AGE_2: 25–39 years (reference category); and AGE_3: 40–49 years. We omitted residence from the multivariate analyses owing to its high correlation with education and wealth. The variable EXPO_RISK takes a value of 1 for women exposed to the risk of pregnancy and 0 for those who are not. We would have used this variable to retain only women at risk of pregnancy when modeling contraceptive use, but in wanting to retain a sufficiently large sample, we decided to use it in the model. We do not use it while modeling intentions for future use. We added the variable SE_NA at the same level as the other dimensions. This variable takes a value of 1 if the respondent is unaware of any side effects and 0 if she is.

To maintain consistency with the framework, we included a cluster-level analysis to model sociocultural and supply-side effects on the psychosocial dimensions. This cluster-level variable portrays the extent to which women have been exposed to FP communication strategies conducted within the geographical area. We used the mean number of media sources from which women in the area had absorbed FP-related messages within the last few months.

### Models

**Fig. 1:**
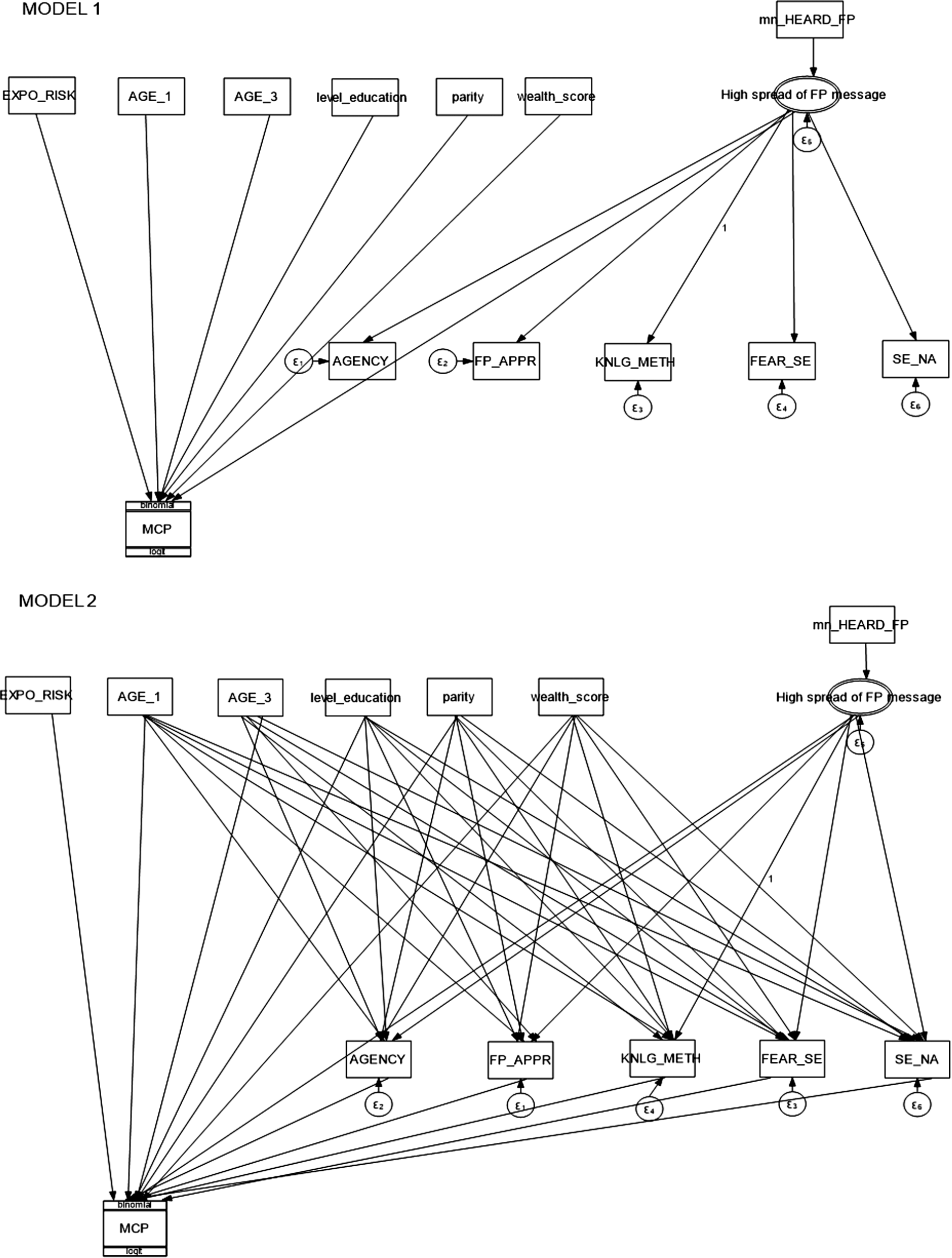
Path diagram of the association between psychosocial dimensions and individual and contextual characteristics

We analyze the associations between psychosocial dimensions, contraceptive use, intention to use, and women’s characteristics. We consider individual and contextual-level characteristics. In Fig. 1, contextual latent variables are represented by double-ringed ovals, while their observed exogenous or anchor variables are the boxes above them. The other socioeconomic (level of education and wealth score) and life-course variables (age and parity) were also exogenously observed variables. Using arrows, we portray the effect of one variable on another. In the first model, we consider the direct action of individual characteristics on modern contraceptive use (MCP) (or intention to use, respectively), while contextual variables are acting on the psychosocial dimensions and contraceptive use. Therefore, in the first model, we controlled for the effect of each of the individual characteristics. In the second model, we added paths from individual characteristics to a psychosocial dimension, and then to modern contraceptive use. As such, we nested model 1 within model 2 and we compared them to determine which better explains the relationships between our variables. We performed a likelihood ratio (LR) test to compare the two models.

For the estimations, we must constrain one coefficient of the multilevel coefficient. We constrained the coefficient between contextual factors and contraceptive knowledge to “1” for two reasons. The first reason is that we believe knowledge to be a more neutral condition that can serve as a reference for interpreting the other dimensions. The second reason—a statistical one—is that it allows better estimations for other coefficients. Therefore, we must keep in mind that the contraceptive knowledge variable is the reference when interpreting the coefficients of the relations between contextual variables and other psychosocial dimensions. We used the variable SE_NA in the models as a control variable.

## Results

### Association between psychosocial dimensions and intention for future use of contraception

Here, we study the association between psychosocial dimensions, individual and contextual characteristics, and women’s intentions concerning contraceptive use. That analysis concerns all married women who are not using contraception. We used the previous two diagrams to produce two models. Table 1 summarizes the results of the two models.

#### Model 1

Older women (40–49 years) appear to have a significantly lower level of intention to use contraception in the future than the reference category (25–39 years). Younger women aged 15–24 years tend to have a higher level of intention to use contraception in the future. Wealth score is also negatively associated with intention for future use. Apart from age and wealth, none of the other variables is associated with the intention to use contraception. We conclude that only age is significantly associated to use contraception in the future.

#### Model 2

Contraceptive approval and agency were significantly associated with intention to use contraception. Age is still associated with intention to use in the same directions as in the first model. However, the coefficient of the younger year group increased while the absolute value of the coefficient of the oldest age group decreased. Wealth score is also significantly associated with intention to use, but its effect is negative. Concerning the relations between individual characteristics and psychosocial dimensions, those in the first age group have less agency (-0.378) and knowledge (-0.927) and tend to be less aware of side effects than the reference category. The older age group tends to have a lower level of approval, agency, and knowledge of SE than the middle group, but no significant difference is evident between the two groups concerning the level of knowledge regarding methods and fear of side effects. Wealth is positively associated with contraceptive knowledge but negatively associated with the fear of side effects. The contextual variable was positively associated with agency but not with contraceptive intention. Considering the relationship between age and intention that was classically studied, we observed approval and agency to have mediation effects. A mediation effect is observed when the addition of the third variable modifies (i.e. decreases or increases) the effects of the independent variable [25] (MacKinnon, Krull, and Lockwood 2000). Indeed, taking approval and agency into account in the relationship between age groups and intention decreases the effect of AGE_3 and increases that of AGE_1, while these two categories are significantly associated with the approval and agency variables.

**Table 1:** Multilevel structural equation modeling of intention for future use of contraception

VARIABLES	Intention to use	FP_APPR		AGENCY	KNLG_METH	FEAR_SE	SE_NA
*Model 1*							
AGE_1 (15–24 years)	0.537***						
	(0.191)						
AGE_3 (25–39 years) **Ref**							
AGE_3 (40–49)	-1.901***						
	(0.225)						
Level of education	-0.013						
	(0.106)						
Parity	0.046						
	(0.039)						
Wealth score	-0.095**						
	(0.038)						
High spread of FP message [EA_ID]	0.676	2.974		1.603	1	0.0484	-0.125
	(0.526)	(2.426)		(1.043)	(0)	(0.071)	(0.084)
***Information criteria***	N = 1,393	*Df = 23*		*AIC = 29,349*		*BIC = 29469.5*	
*Model 2*							
Contraceptive approval (**FP_APPR)**	0.277***						
	(0.029)						
Contraceptive agency (**AGENCY)**	0.070***						
	(0.022)						
Contraceptive Knowledge (**KNLG_METH)**	0.027						
	(0.035)						
Fear of side effects (**FEAR_SE)**	0.019						
	(0.276)						
AGE_1 (15–24 years)	0.767***	-0.812***		-0.378**	-0.927***	-0.049	0.105**
	(0.208)	(0.314)		(0.148)	(0.231)	(0.037)	(0.042)
AGE_3 (25–39 years) **Ref**							
AGE_3 (40–49)	-1.841***	-0.875***		-0.685***	-0.075	-0.0647*	0.088**
	(0.250)	(0.274)		(0.200)	(0.257)	(0.036)	(0.043)
Level of education	-0.138	0.626***		0.251***	0.922***	0.043**	-0.069***
	(0.126)	(0.162)		(0.088)	(0.122)	(0.021)	(0.021)
Parity	0.027	0.031		0.064*	0.036	0.012*	-0.016**
	(0.042)	(0.052)		(0.033)	(0.041)	(0.006)	(0.008)
Wealth score	-0.083**	0.120		0.064	0.201***	0.013	-0.031***
	(0.04)	(0.097)		(0.053)	(0.053)	(0.009)	(0.012)
Do not know any side effect (SE_NA)	0.0211						
	(0.253)						
High spread of FP message in EA	0.0295	3.335		1.785**	1	0.042	-0.122
	(0.212)	(2.306)		(0.876)	(0)	(0.066)	(0.089)
*Information criteria*	*N =* 1,393	*Df = 53*		*AIC = 28863.59*		*BIC = 29141.27*	
Likelihood-ratio test (Model 1 and Model 2)	LR chi2(30) = 545.41			*P*-value = 0.0000			

Women 15–49 years old, married, non-users of modern methods, who know contraception. Notes: *FP = Family planning. Data are from PMA2020 round 6 data collected in Burkina Faso 2018/2019. EA : Enumeration area

SE_NA : Side effect is not available. Df: Degree of freedom; AIC: Akaike Information Criterion; BIC: Bayesian Information Criterion ; LR: Likelihood-ratio

Robust standard errors in parentheses

*** *p* < 0.01, ** *p* < 0.05, * *p* < 0.1

### Association between psychosocial dimensions and contraceptive use

In this second part (Table 2), we study the associations with modern contraceptive use among married women. We do not exclude women who are not exposed to the risk of pregnancy, but we use the variable “exposure to the risk of pregnancy,” for which we control the models.

#### Model 1

Level of education, parity, and exposure to the risk of pregnancy are significantly associated with modern contraceptive use. Indeed, the more women are educated or have children, the more likely it is that they will use modern contraception. Women who have fewer children use modern contraceptives less often than those who have more children. The contextual latent variable has no significant effect (constrained to 1 for contraceptive knowledge) on contraceptive use.

#### Model 2

Among the psychosocial dimensions, contraceptive approval and contraceptive agency are strongly associated with modern contraceptive use. Among individual characteristics, level of education, parity, and exposure to the risk of pregnancy remained significantly associated with modern contraceptive use. We note that the coefficient of education has decreased and become less significant than in model 1. Parity and exposure to the risk of pregnancy also registered a slight decrease in their coefficients but with the same level of significance.

The youngest group (15–24 years) exhibits significantly lower levels of approval, agency, and knowledge than the middle group (25–39 years) but no significant difference regarding fear of side effects. The oldest age group (40–49 years) also exhibits lower levels of approval and agency than the middle group, with no significant difference in knowledge or fear of side effects. The trend according to age group indicates that, compared to the middle group, younger girls tend to lack knowledge about contraception and its side effects while older women exhibit lower approval of contraception. The level of education is significantly and positively associated with approval, agency, knowledge, fear of side effects, and knowledge of SE. Parity is also positively associated with contraceptive agency and fear of side effects. Wealth has no significant impact on contraceptive approval but is positively associated with knowledge of contraception. The contextual variable of support for high fertility has a coefficient significant and positive coefficient with agency.

From the two models, we observed a slight decrease in the coefficient of the level of education, parity, and exposure to the risk of pregnancy when psychosocial variables were added—particularly approval and agency, which appear to be significantly associated with the former individual characteristics (education, parity). We can conclude that some effects of these individual characteristics are mediated by contraceptive approval and agency. The significant reduction in the regression coefficient between education and modern contraceptive use may be explained by the positive effects of approval and agency on contraceptive use.

**Table 2:** Multilevel structural equation modeling of modern contraceptive use

VARIABLES	Modern contraceptive use	FP_APPR	AGENCY		KNLG_METH		FEAR_SE	SE_NA
*Model 1*								
AGE_1 (15–24 years)	-0.216							
	(0.137)							
AGE_3 (25–39 years) **Ref**								
AGE_3 (40–49)	-0.271							
	(0.173)							
Level of education	0.374***							
	(0.083)							
Parity	0.115***							
	(0.027)							
Wealth score	0.056*							
	(0.032)							
Exposure to the risk of pregnancy	2.268***							
	(0.183)							
High spread of FP message [EA_ID]	0.113	2.210	1.148		1		0.046	-0.112
	(0.111)	(1.681)	(0.701)		(0)		(0.060)	(0.069)
*Information criteria*	N = 2,191	*Df = 24*	*AIC =* 43130.5				*BIC = 43267.11*	
*Model 2*								
Contraceptive approval (FP_APPR)	0.167***							
	(0.032)							
Contraceptive agency (AGENCY)	0.048**							
	(0.023)							
Contraceptive Knowledge (KNLG_METH)	0.066*							
	(0.034)							
Fear of side effects (FEAR_SE)	-0.025							
	(0.249)							
AGE_1 (15–24 years)	-0.056	-0.593**	-0.317**		-0.858***		-0.033	0.079**
	(0.136)	(0.282)	(0.128)		(0.185)		(0.032)	(0.037)
AGE_3 (25–39 years) **Ref**								
AGE_3 (40–49)	-0.235	-0.909***	-0.561***		-0.06		-0.037	0.048
	(0.185)	(0.239)	(0.152)		(0.208)		(0.028)	(0.033)
Level of education	0.243**	0.696***	0.326***		0.883***		0.050***	-0.079***
	(0.101)	(0.114)	(0.075)		(0.097)		(0.018)	(0.019)
Parity	0.1***	0.057	0.090***		0.053		0.014**	-0.018**
	(0.029)	(0.052)	(0.029)		(0.035)		(0.006)	(0.007)
Wealth score	0.042	0.119	0.027		0.206***		0.006	-0.022**
	(0.033)	(0.075)	(0.046)		(0.041)		(0.008)	(0.01)
Do not know any side effect (SE_NA)	-0.188							
	(0.257)							
Exposure to the risk of pregnancy	2.233***							
	(0.186)							
High spread of FP message in EA	-0.420*	3.087*	1.651**		1		0.011	-0.0799
	(0.247)	(1.828)	(0.676)		(0)		(0.0609)	(0.0841)
*Information criteria*	*N =* 2,191	*Df = 54*	*AIC = 42439.27*				*BIC =* 42746.64	
Likelihood-ratio test (Model 1 - Model 2)	LR chi2(30) = 751.23		*P*-value = 0.000					

Married women of 15–49 years who know contraception. Notes: *FP = Family planning. Data are from PMA2020 round 6 data collected in Burkina Faso 2018/2019. EA : Enumeration area.

SE_NA : Side effect is not available. Df: Degree of freedom; AIC: Akaike Information Criterion; BIC: Bayesian Information Criterion ; LR: Likelihood-ratio

Robust standard errors in parentheses

*** *p* < 0.01, ** *p* < 0.05, * *p* < 0.1

## Discussion

Beginning from the data on four psychosocial dimensions collected in Round 6 of PMA2020in Burkina Faso, we analyzed their internal relations and their associations with individual and contextual characteristics of women as well as with contraceptive use and intention to use.

The positive effect of knowledge of contraception, approval and agency on contraceptive use in the multivariate analysis confirms the trend we found in a previous study [5]. These relationships are not new, as they portray the idea that favorable assessment, awareness of modern contraceptives, and the ability to perform contraceptive behavior prepare women for modern contraceptive use. Despite the “classical” flavor of this result, these variables are not routinely collected anymore (for approval) or used in analyses (for knowledge of contraceptives, which is “close to universal” based on the knowledge of at least one contraceptive method). Our results emphasize the needfor renewed attention to the concept of knowledge whichgoes beyond ordinary awareness of contraceptive methods. Indeed, the knowledge dimension of access also encompasses information about alternative methods, side effects, management of side effects, and method removal. Other researchers have emphasized the importance of counseling, which can assist a woman in becoming acquainted with various methods and selecting the most suitable and effective one for her [26] (Demir et al., 2020). Additionally, it has been demonstrated that proper counseling can guide a woman from nonuse or traditional methods use toward modern contraceptive use [27] (Ugurlucan et al., 2020).

Our results also reveal the importance of approval as a crucial aspect of psychosocial accessibility particularly in understanding the connections between socioeconomic and life-course variables and contraceptive use. Indeed, the more educated and the older (i.e. middle reproductive age) women are, the more likely they are to approve of FP and exhibit greater agency, which in turn affects contraceptive use. The impact of education on approval has been verified by earlier studies [18, 28, 29] (Ainsworth et al. 1996; Congo 2007; Rossier 2007), but this mediator remains understated in the literature on education and contraception. Irrespective of the use or not of a modern method, we found that younger women were more likely to lack agency and knowledge about contraception. The lack of contraceptive knowledge among young girls has been observed in earlier studies [30, 31] (Bationo 2012; Chandra-Mouli et al. 2014). Older women, while engaging in the same level of contraceptive use as the middle group, tended to have lower levels of contraceptive approval. This result is surprising but is in line with the fact that non-users in the oldest age group are less inclined to use contraception. These results being controlled for parity, education, and wealth, we believe that they may be attributable to this age group’s attachment to more traditional views on contraception.

The impact of parity on approval may be explained by the fact that women who have not reached their ideal number of children may not be amenable to contraceptive use. Many studies and reports have shown that women start using contraceptives after having some number of children (the average number of children at first use is 1.5 in urban areas and 3.3 in rural areas) [32] (PMA/Burkina 2020). This result is also confirmed by a study showing that nulliparous married women tend to engage in lower levels of contraceptive use [33] (de Vargas Nunes Coll et al. 2019).

Turning to contraceptive agency, our results indicate that younger women are more likely to lack the ability to avail of contraception than women 25 − 39 years group. This result was observed in a study in which many adolescents in low- and middle-income countries wish to postpone pregnancy but lack the agency or resources to implement their desires [31] (Chandra-Mouli et al. 2014). On the other hand, the impact of education on contraceptive agency, and that of wealth on contraceptive knowledge, are also important in explaining the low use of contraceptives among poor and non-educated women. This result is also confirmed by the strong association between education and knowledge of methods. In other words, underprivileged women tend to lack agency and access to useful information (in terms of knowledge of methods) that can make them more amenable to the approval and use of FP.

Our results suggest that even if side effects can deter some non-users, other women who are users—when committed to avoiding pregnancy—manage to withstand the negative feelings associated with these side effects. However, even if these fears do not seem to influence contraceptive use in our analysis, we must take care as they may sometimes supersede the intentions of women who are not wholly committed to pregnancy avoidance. In other words, fears of side effects can make a difference for women whose intentions of avoiding pregnancy are weak. We could not test this hypothesis here, as no data on the strength or ambivalence of pregnancy avoidance desires were collected in mainstream contraceptive surveys. Along the same line, as household wealth is correlated with residence, the geographical access and availability of health services must also be considered in explaining this difference. In other words, the supply-related dimensions of accessibility seem to interact with the existence of these psychosocial obstacles to contraceptive use. Further research on this topic is required.

Regarding the contextual variable (i.e. the dissemination of FP-related messages in the community), it tends to impact contraceptive agency compared to contraceptive knowledge. However, we observed a limited effect of FP messages in the local context on contraceptive use, approval, and knowledge. We do not have enough information to fully explain this limited effect. The specificities of the FP messages (i.e. content, media, frequency) in Burkina Faso in the year preceding the survey may help explain the non-relationship observed here, which contrasts with the results of several studies [34]. This study faced several limitations, mainly relating to the data available. Indeed, we include women who are unaware of any side effects based on the assumption that they have no fears of side effects. That assumption may be risky because non-users may be less likely to know the side effects. This fact may explain the absence of any observable relationship between fear of SE and other variables. However, excluding these women would have resulted in less than half of the total sample. Therefore, these analyses must be tested on a large sample wherein all respondents are aware of at least one side effect and have stated the level of fear they experience concerning side effects. Other limitations are related to the endogeneity of variables like knowledge and fear of side effects with contraceptive use. Users may be much more aware of other methods and side effects than non-users. Social desirability bias may also affect the likelihood that users will say they approve of FP and are familiar with side effects, while non-users may be more tempted to say that they ignore contraception methods and their potential side effects. Intentions to use contraception in the future may also be influenced by older users’ experiences, which we did not consider. We also lack data on the types of FP messaging that may have spread throughout the country during the year preceding the survey. Future studies must endeavor to unpack these messages to understand their effects on psychosocial dimensions of access, such as contraceptive knowledge, approval, agency, and fear of side effects. Despite this limitation, our results can help improve knowledge about cognitive and psychosocial access to contraception.

## Conclusion

Here, we have measured the effects of psychosocial dimensions of access on contraception use and intentions to use in the future, while considering individual and contextual characteristics. Even if we encounter some limitations, the results are enlightening to a considerable extent. Knowledge, approval, and agency are directly and indirectly related (as mediators of socioeconomic variables) to contraceptive use and intention to use. However, fear of side effects does not make a significant difference here, which is probably linked to measurement issues.

The results discussed above highlight several conclusions on aspects that should be considered by both researchers and practitioners. Researchers are invited to pay greater attention to psychosocial dimensions to improve their understanding of access to contraception and the remaining obstacles to contraceptive use in low-income countries. Constructing and analyzing detailed items that capture the variety of psychosocial factors will help to measure and understand the psychosocial effect on contraceptive use behavior. Particularly, aspects such as knowledge, approval, and fear of side effects remain rarely investigated in comparison to agency. Those efforts must not neglect to measure sociocultural norms and perceptions, which may impact most individual psychosocial dimensions. Measurement of the strength of fertility intentions should also be considered, as some psychosocial factors may impact contraceptive use in women and couples whose motivation to avoid pregnancy is weak.

For practitioners, policymakers, and intervention designers, on top of current efforts to promote accessibility on the supply side, efforts must be made to reduce inequality of access to formal information on contraceptives and their side effects among younger, poorer, and less educated women. Moreover, actions must be oriented toward the dissemination of FP-related messaging that enhances women’s approval of contraception and their ability to discuss, negotiate, or make decisions regarding childbearing and FP issues. Therefore, to be effective, FP messages must consider local representations of fertility, particularly those spread through religions that discourage the use of modern contraceptives. At the same time, in a human rights-based approach (WHO 2014), all practitioners involved in FP programs must be reminded that just as individuals have the right to avail of a method, they also have the right to refuse it. Potential clients will ultimately decide whether they will adopt a method. Unmet needs and accessibility indicators are thus important for *planning* the delivery of contraceptive services to all women in need (i.e. women who wish to avoid pregnancy), but the goal for public powers should never be to attain 100% accessibility and 0% unmet need. Women and couples who express the desire to avoid pregnancy have the right to adhere—after being informed of their options—to a vision of fertility regulation other than that proposed by the global health model.

## Data Availability

Data available publicly available on https://fr.pmadata.org/.

## References

[1] Ministère de la santé. (2017). *Plan National d’Accélération de Planification Familiale du Burkina Faso 2017–2020*.

[2] INSD & MACRO (2012). Enquête Démographique et de Santé 2010.

[3] Skiles MP, Cunningham M, Inglis A, Wilkes B, Hatch B, Bock A, Barden-O’Fallon J (2015). The effect of access to contraceptive services on injectable use and demand for family planning in Malawi. Int Perspect Sex Reproductive Health.

[4] Choi Y, Fabic MS, Adetunji J (2016). Measuring Access to Family Planning: conceptual frameworks and DHS Data. Stud Fam Plann.

[5] Zan LM. (2021). Planification familiale au Burkina Faso dans la décennie 2010–2019: Rôle des dimensions cognitives et psychosociales dans l’accès [PhD Thesis]. University of Geneva.

[6] Dehlendorf C, Fox E, Sharma AE, Zhang J, Yang S, Centola D. (2020). Birth Control Connect: A randomized trial of an online group to disseminate contraceptive information. *Contraception*. 10.1016/j.contraception.2020.01.014 PMC7234911 32032641

[7] Gilliam ML, Warden M, Goldstein C, Tapia B (2004). Concerns about contraceptive side effects among young latinas: a focus-group approach. Contraception.

[8] Pazol K, Zapata LB, Dehlendorf C, Malcolm NM, Rosmarin RB, Frederiksen BN (2018). Impact of Contraceptive Education on knowledge and decision making: an updated systematic review. Am J Prev Med.

[9] Rogers EM. (2010). *Diffusion of Innovations, 4th Edition*. Simon and Schuster.

[10] Casterline JB, Perez AE, Biddlecom AE. (1997). Factors underlying unmet need for family planning in the Philippines. *Studies in family planning*, 173–191. 9322334

[11] Miller WB (1986). Why some women fail to use their contraceptive method: a psychological investigation. Fam Plan Perspect.

[12] Sedlander E, Bingenheimer JB, Thiongo M, Gichangi P, Rimal RN, Edberg M, Munar W (2018). They destroy the reproductive system: exploring the belief that modern contraceptive use causes infertility. Stud Fam Plann.

[13] Bongaarts J, Bruce J. (1995). The causes of unmet need for contraception and the social content of services. Stud Fam Plann, 57–75. 7618196

[14] Sondo B, Sya D, Paré R, Kouanda S, Savadogo L (2001). L’utilisation Des méthodes contraceptives par les Mossi d’un district sanitaire rural de Kaya, Burkina Faso. Cahiers d’études et de recherches francophones/Santé.

[15] Rossier C, Bernardi L (2009). Social interaction effects on fertility: intentions and behaviors. Eur J Population/Revue européenne de Démographie.

[16] Islam S, Hasan M (2016). Women knowledge, attitude, approval of family planning and contraceptive use in Bangladesh. Asia Pac J Multidisciplinary Res.

[17] Cleland JG, Ndugwa RP, Zulu EM (2011). Family planning in sub-saharan Africa: Progress or stagnation?. Bull World Health Organ.

[18] Rossier C (2007). Attitudes towards abortion and contraception in rural and urban Burkina Faso. Demographic Res.

[19] Bamiwuye SO, De Wet N, Adedini SA (2013). Linkages between autonomy, poverty and contraceptive use in two sub-saharan African countries. Afr Popul Stud.

[20] Ghose B, Feng D, Tang S, Yaya S, He Z, Udenigwe O, Ghosh S, Feng Z. (2017). Women’s decision-making autonomy and utilisation of maternal healthcare services: results from the Bangladesh Demographic and Health Survey. BMJ open. 7(9);e017142. 10.1136/bmjopen-2017-017142 PMC5595179 28882921

[21] Kabeer N (1999). Resources, agency, achievements: reflections on the measurement of women’s empowerment. Dev Change.

[22] Sapin M, Spini D, Widmer E. Les parcours de vie: De L’adolescence Au grand âge. Volume 39. Collection le savoir suisse; 2007.

[23] James-Hawkins L, Peters C, VanderEnde K, Bardin L, Yount KM (2018). Women’s agency and its relationship to current contraceptive use in lower-and middle-income countries: a systematic review of the literature. Glob Public Health.

[24] Acock AC. Discovering structural equation modeling using Stata. Stata Press Books; 2013.

[25] MacKinnon DP, Krull JL, Lockwood CM (2000). Equivalence of the mediation, confounding and suppression effect. Prev Sci.

[26] Demir O, Ozalp M, Sal H, Aran T, Osmanagaoglu MA (2021). Evaluation of the frequency of coitus interruptus and the effect of contraception counselling on this frequency. J Obstet Gynaecol.

[27] Ugurlucan FG, Demir O, Tas S, Dural O, Yasa C, Yalcin O (2020). Contraception counselling during gynecology visit—does a questionnaire help?. Ginekologia Polska.

[28] Ainsworth M, Beegle K, Nyamete A (1996). The impact of women’s schooling on fertility and contraceptive use: a study of fourteen sub-saharan African countries. World Bank Econ Rev.

[29] Congo Z. (2007). *Les facteurs de la contraception au Burkina Faso au tournant du siècle: Analyse des données de l’enquête démographique et de santé 1998/1999*.

[30] Bationo BF (2012). Les relations entre les professionnels de santé et les jeunes filles Au Burkina Faso. Agora débats/jeunesses.

[31] Chandra-Mouli V, McCarraher DR, Phillips SJ, Williamson NE, Hainsworth G (2014). Contraception for adolescents in low and middle income countries: needs, barriers, and access. Reproductive Health.

[32] PMA/Burkina. (2020). *PMA BURKINA FASO Results from Phase 1 baseline survey, December 2019—February 2020*.

[33] de Coll VN, Ewerling C, Hellwig F, de Barros AJD (2019). Contraception in adolescence: the influence of parity and marital status on contraceptive use in 73 low-and middle-income countries. Reproductive Health.

[34] Mwaikambo L, Speizer IS, Schurmann A, Morgan G, Fikree F (2011). What works in family planning interventions: a systematic review. Stud Fam Plann.

